# Acute Hypertriglyceridemia in Patients with COVID-19 Receiving Parenteral Nutrition

**DOI:** 10.3390/nu13072287

**Published:** 2021-07-01

**Authors:** Gema Villa López, Maria Angeles Valero Zanuy, Ivan González Barrios, Maria Maíz Jiménez, Pilar Gomis Muñóz, Miguel León Sanz

**Affiliations:** 1 Clinical Nutrition Unit, Department of Endocrinology and Nutrition, Hospital Universitario 12 de Octubre, Avenida de Córdoba, s/n, 28041 Madrid, Spain; g.villa@salud.madrid.org (G.V.L.); mariaangeles.valero@salud.madrid.org (M.A.V.Z.); mariairene.maiz@salud.madrid.org (M.M.J.); 2 Department of Pharmacy, Hospital Universitario 12 de Octubre, 28041 Madrid, Spain; igbarrios@salud.madrid.org (I.G.B.); pgomis.hdoc@salud.madrid.org (P.G.M.)

**Keywords:** COVID-19, acute respiratory distress syndrome, hypertriglyceridemia, fatty acids, lipidic emulsions, parenteral nutrition

## Abstract

Hypertriglyceridemia is a metabolic complication associated with parenteral nutrition (PN). It is unknown if patients with acute respiratory distress syndrome (ARDS) secondary to COVID-19 are more at risk. Our aim was to describe the incidence, risk factors and clinical impact of hypertriglyceridemia in critically ill patients with ARDS-COVID-19 receiving PN. We designed a cohort study of patients with ARDS-COVID-19 infection that required admission to critical care units and nutritional support with PN. Individual PN prescriptions for macronutrients and insulin were provided. Lipid emulsion contained fish oil (SMOFlipid^®^ or Lipoplus^®^). Hypertriglyceridemia was defined as plasma levels above 400 mg/dL. Eighty-seven patients, 66.6% men, 60.1 ± 10.8 years old, BMI 29.1 ± 5.6 kg/m^2^, 71% of whom received lopinavir/ritonavir, 56% received Propofol and 55% received Tocilizumab were included. The incidence of hypertriglyceridemia was 37 × 100 patient-days with PN. This complication was more frequent in obese patients (OR 3.34; 95% CI, 2.35–4.33) and in those treated with lopinavir/ritonavir (OR 4.98; 95% CI, 3.60–6.29) or Propofol (OR 2.45; 95% CI, 1.55–3.35). Total mortality was 33.3%, similar between the type of lipid emulsion (*p* = 0.478). On average, patients with hypertriglyceridemia had a longer requirement of PN compared to the group without elevated triglycerides (TG), probably because of their longer survival (*p* = 0.001). TG higher than 400 mg/dL was not a protective factor for mortality (OR 0.31; 95% CI, 0.01–1.30). In conclusion, the incidence of hypertriglyceridemia was 37 × 100 patient-days with PN. The risk of this complication is associated with obesity and the use of lopinavir/ritonavir or Propofol.

## 1. Introduction

An outbreak of coronavirus disease 2019 (COVID-19) was described in Wuhan (China) in December of 2019. The disease spread globally and has become a significant threat to international health. COVID-19 disease has a variable clinical spectrum ranging from asymptomatic cases to patients with respiratory failure and acute respiratory distress syndrome (ARDS), septic shock and multiorgan failure. The elderly and those with comorbidities are more likely to present with severe forms of COVID-19 disease [1].

Approximately 20% of individuals with COVID-19 disease present with ARDS and require admission to intensive care units (ICU) [2].

Enteral nutrition (EN) is the preferred form of nutritional support in critical patients in the ICU. However, parenteral nutrition (PN) should be considered when EN is not feasible or patients are unable to meet the energy and protein requirements by the enteral route alone [3,4]. Lipid emulsions are a component of PN which provide essential fatty acids and avoid glucose excess as a non-protein energy source [5]. Nevertheless, several complications secondary to the use of lipid emulsions have been described, including lipid profile alterations such as hypertriglyceridemia, disrupted immune responses, and liver disease [6]. Hypertriglyceridemia associated with PN is multifactorial. Several risk factors have been associated with hypertriglyceridemia, including excess glucose or a type of lipid emulsion in PN, history of obesity, diabetes, enterocutaneous fistula, sepsis or multiorgan failure or the use of certain drugs [7]. The acute inflammatory response in COVID-19-related ARDS may alter lipid clearance in patients with PN, contributing to hypertriglyceridemia. In addition, some drugs may also interfere with lipid metabolism and increase the risk. It is unknown what role the altered lipid profile plays, or its clinical evolution. Typically, hypertriglyceridemia has been associated with a decreased capacity in pulmonary diffusion, altered immune response and acute pancreatitis, especially if plasma triglyceride (TG) levels exceed 1000 mg/dL [8]. 

In routine clinical practice, with adjusted contributions of carbohydrates and lipids in PN in critically ill patients, it is rare to observe hypertriglyceridemia. However, we observed plasma hypertriglyceridemia in many patients in ICU with COVID-19-related ARDS, when they required PN during the coronavirus outbreak in 2020. Therefore, we designed a prospective study to analyze the incidence, risk factors and clinical relevance of hypertriglyceridemia in this group of patients.

## 2. Materials and Methods

The study was a prospective, observational analysis of a cohort of critically ill COVID-19 patients that included adult inpatients (≥18 years old) diagnosed with COVID-19-related ARDS [9] that required intubation and mechanical ventilation. All adult patients with this condition, admitted to a critical care unit run by the Anesthesiology Department during the first wave of the pandemic were included if they were expected to need PN for at least 5 days. Patients with a clinical history of inborn error of lipid metabolism were excluded. PN was indicated when enteral access was not feasible, such as in the presence of adynamic ileus, gastrointestinal dysfunction, or in those who required prone positioning in order to improve the pO_2_/FiO_2_ ratio when enteral nutrition was insufficient [10]. Patients finished the study when they achieved oral or enteral complete tolerance or when they died during hospitalization.

The PN formulation was individualized. Caloric requirements were calculated according to age, sex, weight and height with Harris–Benedict formula and multiplying by a stress illness factor of 1.3. In obese patients, we used the adjusted weight to calculate caloric requirements using the following formula:

Adjusted weight = (Actual weight − Ideal weight) × 0.25 + Ideal weight

PN composition was adjusted according to the recommendations of the Society of Critical Care Medicine (SCCM) and American Society for Parenteral and Enteral Nutrition (ASPEN) for the nutrition support of critically ill patients [11]. Two different lipid emulsions were used: SMOFlipid^®^ (Fresenius Kabi), which contains a mix of 30% soybean oil, 30% MCT oil, 25% olive oil and 15% fish oil; or Lipoplus^®^ (Braun), which contains a mix of 40% soybean oil, 50% MCT oil and 10% fish oil. Electrolytes were adjusted according to the patient’s daily needs. All PN bags provided daily trace elements (Supliven^®^, Fresenius) and vitamins (Cernevit^®^, Baxter). The final volume of the PN was approximately 1 mL/kcal, unless the patient needed water restriction. The infusion of a 3-in-1 PN included a 1.2 µm filter to limit the infusion of unwanted particles, as recommended by the Federal Drug Administration [12]. All PN bags were tailor-made in the compounding facilities of the Department of Hospital Pharmacy of our institution and administered for 24 h. Therefore, we did not use ready-to-use standard bags.

If patients presented hyperglycemia, regular insulin was initially administered in the PN bag at a rate of 1 unit per 10 g of glucose, along with supplemental subcutaneous regular insulin, to maintain a plasma glucose range between 140 and 180 mg/dL. Two-thirds of the subcutaneous regular insulin needed to keep blood glucose in range in the previous 24 h was added the next day to the PN.

The variables of the study were age (years), sex (woman/man), history of diabetes mellitus, dyslipidemia, or obesity (yes/no), body mass index (BMI defined as weight in kilograms /height^2^ in meters), average composition of the PN in kilocalories (kcal), amino acids in grams (g), glucose (g), lipids (g), insulin dose (units/day) and need for treatment with drugs (yes/no): Propofol^®^ (Fresenius Kabi), Remdesivir (off label, Gilead Sciences), Tocilizumab (Roactemra, Roche) and Lopinavir/ritonavir (Kaletra^®^, Abbvie Farmaceutica). When Propofol was needed, we adjusted the amount of lipids in PN resting at 0.1 g per mL of Propofol administered. We also recorded the days of duration of the PN and mortality in hospital.

Per protocol, serum biochemical parameters (albumin and C-reactive protein) were evaluated daily and TG weekly, depending on the starting day of PN administration. C-reactive protein and albumin values were determined in serum by immunoturbidimetry technique, whereas plasma TG levels were analyzed by spectrometry using a colorimetric enzymatic method. Both were determined on the Roche/Hitachi Cobas 8000 autoanalyzer module c701 (Roche Diagnostics^®^).

Hypertriglyceridemia was defined if the patient had at least a plasma TG value above 400 mg/dL. The incidence of hypertriglyceridemia was calculated as the number of patients with plasma TG ≥ 400 mg/dL per 100, divided by the total number of patient-days of PN. Lipid emulsions were withheld from the PN in patients with hypertriglyceridemia. The resulting decrease in energy provided in the PN bag was not compensated by increases in glucose or amino acids. These patients received 50 g of lipids weekly to avoid the development of fatty acid deficiency [13]. Lipids were administered mixed in the PN.

The study was carried out following the ethical guidelines established for studies in patients with COVID-19; no diagnostic or therapeutic intervention other than standard practice has been carried out. The Institutional Review Board (IRB) was informed of the research on the study. Descriptive statistics were assessed, calculating the mean and standard deviation for quantitative variables and relative frequencies for qualitative variables. Comparisons between binary datasets used two sample t-tests or chi-squared tests to compare patients with normal plasma TG levels and patients with plasma TG levels above 400 mg/dL. We used a logistic regression to estimate the odds ratio (OR) of having hypertriglyceridemia for the different risk factors with its corresponding 95% confidence interval (95% CI). Data were processed using SPSS version 18.0. A *p* value ≤ 0.05 was considered statistically significative. 

## 3. Results

The study cohort included 87 patients: 66.7% were men (59.1 ± 11.4 years, 28.4 ± 4.7 kg/m^2^) and 33.3% were women (62.2 ± 9.5 years, 30.5 ± 6.9 kg/m^2^); 24.1% had a diagnosis of diabetes mellitus; 33.3% had dyslipidemia; and 28.7% had obesity, defined as BMI ≥ 30 kg/m^2^. All patients needed mechanical ventilation with a pO_2_/FiO_2_ ratio between 75 and 317. Results of the Harris–Benedict equation estimated basal energy expenditure ranged between 1111 and 1789 kcal/day. Serum albumin and C reactive protein levels were 2.9 ± 0.4 g/dL and 15.0 ± 12.6 g/dL, respectively. Fifty-six percent of patients received an individualized dose of Propofol according to their needs.

After starting PN, thirty-two patients had at least one sample plasma TG value above 400 mg/dL (incidence rate of hypertriglyceridemia of 37 × 100 patient-days with PN). The mean value of plasma TG in the group without and with hypertriglyceridemia was 219.9 mg/dL ± 68.9 mg/dL and 540.6 mg/dL ± 122.2 mg/dL, respectively. Patients with hypertriglyceridemia were younger and weighed more than those with normal plasma TG levels (Table 1). We did not find any correlation of CRP levels with hypertriglyceridemia. A potential explanation is that the distribution of CRP values was very wide (13.4 ± 13.0 vs. 17.7 ± 11.8 g/dL, hypertriglyceridemia and normotriglyceridemia, respectively). Acute pancreatitis was not suspected in patients with hypertriglyceridemia. Therefore, we did not determine pancreatic parameters (such as amylase or lipase).

More patients in the hypertriglyceridemic group received lopinavir/ritonavir (*p* = 0.009) and Propofol (*p* = 0.048). Patients with or without hypertriglyceridemia received homogeneous PN in terms of composition and insulin dose on the first day of nutrition support (Table 2). Patients received 3.2 ± 0.7 g/kg/day of glucose. The PN solution provided lipids such as SMOFlipid^®^ in 66.6% of patients. The average lipid infusion rate the day before the diagnosis of hypertriglyceridemia was approximately 0.04 g/kg/h. Of the 87 patients studied, 69 (79.3%) received insulin (34.1 ± 15.6 ui/day). Table 3 shows the lipid dose from PN and Propofol as well as total lipid dose from long-chain TG the day before the diagnosis of hypertriglyceridemia. The range of total lipids was very wide (0–80 g/day) because three patients did not receive lipids in PN due to an elevation of PN-related liver enzymes. The Propofol range was also very wide (0–30 mL/h) because some patients had difficultly controlling sedation. Patients diagnosed with hypertriglyceridemia had a longer requirement of PN (*p* = 0.001). Biochemical parameters were similar in both groups (data not shown). All patients with hypertriglyceridemia were treated with lipid emulsions of the PN, observing that TG concentrations gradually normalized (<400 mg/dL).

Table 4 shows the adjusted OR and its corresponding 95% CI for hypertriglyceridemia and each variable analyzed. Risk factors for hypertriglyceridemia were obesity (OR 3.34; 95% CI, 2.35–4.33) and the use of Lopinavir/ritonavir (OR 4.98; 95% CI, 3.60–6.29) or Propofol (OR 2.45; 95% CI, 1.55–3.35). 

**Figure 1:**
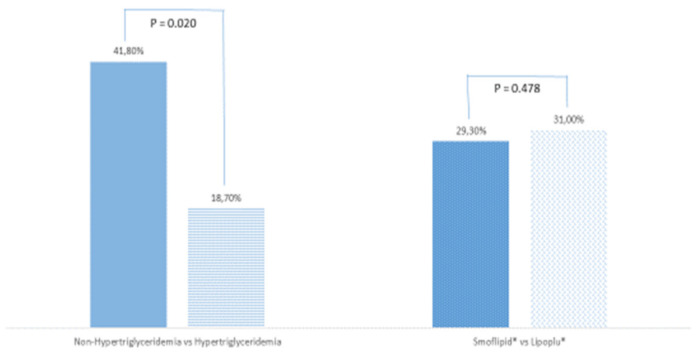
Mortality according to plasma triglyceride levels and lipid emulsion used. Non-HyperTG: patients with normal plasma triglycerides levels; HyperTG: patients with plasma triglyceride levels above 400 mg/dL. *p*-value is significant for values equal to or less than 0.05.

Total mortality was 33.3% (29 patients), especially in patients with normal plasma TG levels (*n* = 23, 41.8%) vs. patients with hypertriglyceridemia (*n* = 6, 18.7%) (*p* = 0.020) (Figure 1). However, TG higher than 400 mg/dL was not a protective factor for mortality (OR 0.31, 95% IC 0.01–1.30). There were no differences in mortality between patients receiving SMOFlipid^®^ (*n* = 17, 29.3%) or Lipoplus® (*n* = 9, 31.0%) (*p* = 0.478). 

## 4. Discussion

Hypertriglyceridemia is a metabolic complication described in patients receiving PN. The frequency is variable, ranging from 6% to 60% [14,15]. The cut-off point defines its definition, the characteristics of the studied population and the composition of the PN. ASPEN suggests that the risk of adverse effects is greatest when plasma TG levels exceed 400 mg/dL [16]. Considering this cut-off point, in our study, the incidence of hypertriglyceridemia was 37 × 100 patient-days with PN. 

This complication is related to different factors. Firstly, the patients’ characteristics such as the need for admission to the ICU and the presence of diabetes, dyslipidemia or obesity were key [17,18]. In our study, 100% had COVID-19-related ARDS requiring mechanical ventilation, and 24.1%, 33.3% and 28.7% were diagnosed with diabetes, dyslipidemia or obesity, respectively. Secondly, hypertriglyceridemia has been linked to the composition of PN. The factors involved are the excess of glucose supply and the type of lipid emulsion used, especially the dose and the source of lipids [19]. Lipid emulsions, particularly those derived from soybean oil, are associated with an increase in plasma TG [20]. In healthy individuals, lipid particles are hydrolyzed by the action of lipoprotein lipase, located at the level of the capillary wall of the adipose and muscular tissue. Under the action of this enzyme, fatty acids are released and used as an energy source, stored in the adipose tissue or metabolized by the liver. The clearing capacity of the lipoprotein lipase depends on the amount of lipids provided, the length of the fatty acid chain, and the phospholipid/TG ratio of the emulsion [21]. An intravenous lipid infusion rate greater than 0.11g/kg/h can saturate the enzyme’s capacity [22]. Medium-chain TG [23], long-chain TG derived from olive oil [24], and fish oil [25] are cleared more rapidly than LCT derived from soybean oil. It has been shown that 20% lipid emulsions have a lower phospholipid/TG ratio than 10% lipid emulsions, and therefore are metabolized faster [26].

Several strategies have been described to try to minimize the risk of hypertriglyceridemia in patients receiving PN. First, an early diagnosis should be made by monitoring plasma lipid levels once a week, because their concentration is an indirect indicator of clearance [27]. Secondly, the glucose supply to the PN must be adjusted. Our patients received an amount of glucose according to international guidelines. Thirdly, consideration should be given to the amount, lipid source and phospholipid/TG ratio. In our study, the average lipid infusion rate the day before the diagnosis of hypertriglyceridemia was approximately 0.04 g/kg/h, and all patients received SMOFlipid^®^ or Lipoplus^®^ 20%. These intravenous lipid emulsions contain less amount of soybean oil, by using fish oils in combination with other sources of TG (SMOFlipid^®^, 30% soybean oil, and Lipoplus^®^, 40% soybean oil). Therefore, they are associated with a reduced inflammatory response and fewer metabolic abnormalities. Although part of these adverse effects may be due to the high percentage of linoleic acid in soybean oil, it must be taken into account that it is an essential nutrient. For this reason, the lipid emulsions most used today combine different lipid sources that maintain the supply of linoleic and alpha-linolenic acid, but modify their omega 6:omega 3 ratio to improve their safety profile. Although some cases of fat overload syndrome have been described with these formulas, its frequency is lower than with soybean oil [28]. Finally, other factors described to be associated with hypertriglyceridemia are drugs [29]. In our case, 56.3% of the cohort received Propofol, with individualized adjusted dose. This could mean that the total amounts of n-6 lipids received by each patient were different. However, the amount of SMOFLipid^®^ or Lipoplus^®^ was also decreased according to the Propofol administered, and in that way, the amount, of n-6 lipids were decreased and more comparable among patients. In addition, in patients infected with COVID-19, the use of Tolicilizumab [30]. and Lopinavir/ritonavir [31] has been described to increase plasma TG levels. In our study, 55.2% and 71.2% of patients had been prescribed these drugs, respectively. Additionally, IV levocarnitine can moderately decrease TG concentrations in patients receiving PN, but the effect is modest [32] and its use is not approved in our country for this indication.

The clinical relevance of hypertriglyceridemia secondary to PN is not well known. It has been associated with increased morbidity and fat overload syndrome [33]. None of our patients presented this syndrome, although we cannot exclude that hypertriglyceridemia contributed to the deterioration of lung function. The plasma TG level was normalized when lipids were discontinued from the PN. Additionally, it has been described in the literature that higher TG levels are associated with mortality [34]. Contrary to these findings, in our study, mortality was higher in the group without hypertriglyceridemia. This can be explained because patients with hypertriglyceridemia received more days of PN on average because their survival rate was longer compared to patients without TG elevations.

Our study has some limitations. Firstly, the interpretation of our findings might be limited by the sample size. Secondly, not all laboratory TG levels were determined in all patients at the same time. Thirdly, although the lipid amount administered took into consideration the use of Propofol, these patients received more lipids and higher n-6 lipid proportions. Finally, those who died earlier had less time with PN to develop hypertriglyceridemia. This could explain why the total mortality was higher in patients with normal TG levels.

## 5. Conclusions

Hypertriglyceridemia is a metabolic complication in patients receiving PN. Intravenous lipid emulsions containing fish oil reduce this complication by accelerating TG clearance. In patients with COVID-19-related ARDS receiving PN, it is important to monitor plasma TG levels and to check the amount of glucose and lipids when lipid infusion is initiated, either as a vehicle for a drug or as an energy source.

## Figures and Tables

**Table 1:** Population characteristics.

	Total (*n* = 87)	Non-HyperTG (*n* = 55)	HyperTG (*n* = 32)	*p*-Value
Age (years)	60.1 ± 10.8	63.5 ± 10.0	54.2 ± 9.7	0.000
Women, *n* (%)	29 (33.3%)	20 (36.3%)	9 (28.1%)	0.224
Men, *n* (%)	58 (66.6%)	35 (63.3%)	23 (71.9%)	0.224
BMI (kg/m^2^)	29.1 ± 5.6	28.7 ± 4.0	31.3 ± 7.2	0.016
Obesity, *n* (%)	25 (28.7%)	12 (21.8%)	13 (40.6%)	0.051
Dyslipidemia, *n* (%)	29 (33.3%)	17 (30.9%)	12 (37.5%)	0.308
Type 2 diabetes, *n* (%)	21 (24.1%)	13 (23.6%)	8 (25.0%)	0.509
Tocilizumab, *n* (%)	48 (55.2%)	28 (50.9%)	20 (62.5%)	0.446
Lopinavir/ritonavir, *n* (%)	62 (71.2%)	33 (60.0%)	29 (90.6%)	0.009
Remdesivir, *n* (%)	5 (5.7%)	4 (7.3%)	1 (3.1%)	0.321
Propofol, *n* (%)	49 (56.3%)	26 (47.3%)	23 (71.9%)	0.048

Data are the mean ± standard deviation or number (%). BMI: body mass index; Non-HyperTG: patients with normal plasma triglycerides levels; HyperTG: patients with plasma triglyceride levels above 400 mg/dL; *p*-value is significant for values equal to or less than 0.05, which represents the difference between Non-HyperTG patients and HyperTG patients.

**Table 2:** Initial composition of macronutrients and parenteral nutrition duration.

	Total (*n* = 87)	Non-HyperTG (*n* = 55)	HyperTG (*n* = 32)	*p*-Value
Calories (kcal/kg/day)	26.9 ± 4.0	26.3 ± 4.0	28.1 ± 3.8	0.591
Amino acids (g/kg/day)	1.3 ± 0.2	1.3 ± 0.2	1.4 ± 0.1	0.099
Glucose (g/kg/day)	3.2 ± 0.7	3.0 ± 07	3.4± 0.6	0.311
Lipids (g/kg/day)	0.9 ± 0.2	0.9 ± 0.1	0.9 ± 0.2	0.808
Insulin dose (iu/day)	34.1 ± 15.6	32.6 ± 13.2	36.3 ± 18.9	0.346
Duration PN (days)	8.5 ± 4.6	7.2 ± 4.2	10.7 ± 4.6	0.001

Data are the mean ± standard deviation or number (%). PN: parenteral nutrition; Non-HyperTG: patients with normal plasma triglycerides levels; HyperTG: patients with plasma triglyceride levels above 400 mg/dL; *p*-value is significant for values equal or less than 0.05, which represents the difference between Non-HyperTG patients and HyperTG patients.

**Table 3:** Lipid dose from PN and Propofol as well as total lipid dose from long-chain TG the day before the diagnosis of hypertriglyceridemia.

	Non-HyperTG (*n* = 55)	HyperTG (*n* = 32)	*p*-Value
Lipids PN (g/day)	55.0 ± 12.7	56.6 ± 21.1	0.670
Lipids PN (g/kg/day)	0.8 ± 0.2	0.8 ± 0.3	0.636
Lipids PN + Propofol (g/day)	63.2 ± 16.2	78.9 ± 27.8	0.004
Lipids PN + Propofol (g/kg/day)	0.9 ± 0.2	1.1 ± 0.4	0.000
LCT (g/day)	43.6 ± 15.1	57.4 ± 25.7	0.007
LCT (g/kg/day)	0.6 ± 0.2	0.8 ± 0.4	0.016

Data are the mean ± standard deviation. PN: parenteral nutrition; Non-HyperTG: patients with normal plasma triglycerides levels; HyperTG: patients with plasma triglyceride levels above 400 mg/dL; *p*-value is significant for values equal or less than 0.05, which represents the difference between Non-HyperTG patients and HyperTG patients.

**Table 4:** Risk factors associated with the development of hypertriglyceridemia (>400 mg/dL) according to a logistic regression.

	OR	CI (95%)
Age (≥65 years/<65 years)	2.52	1.16–5.46
Sex (male/female)	1.64	0.98–2.30
Obesity (IMC ≥ 30 kg/m^2^/<30 kg/m^2^)	3.34	2.35–4.33
Dyslipidemia (yes/no)	1.41	0.69–2.13
Type 2 diabetes (yes/no)	0.88	0.06–1.80
Type of lipids PN (SMOFlipid^®^/Lipoplus^®^)	1.10	0.44–1.76
Tocilizumab (yes/no)	1.19	0.31–2.07
Lopinavir/ritonavir (yes/no)	4.98	3.60–6.29
Remdesivir (yes/no)	0.54	0.01–2.54
Propofol (yes/no)	2.45	1.55–3.35

OR: odds ratio; CI: confidence interval; PN: parenteral nutrition.

## Data Availability

Not applicable.
